# Supplementary material for: A local-ingredients-based supplement is an alternative to corn-soy blends plus for treating moderate acute malnutrition among children aged 6 to 59 months: A randomized controlled non-inferiority trial in Wolaita, Southern Ethiopia
Source: PLoS One. 2021 Oct 28;16(10):e0258715. doi: 10.1371/journal.pone.0258715 (PMC8553037; doi:10.1371/journal.pone.0258715)
Supplement: Attachment — Submitted filename: PONE-D-20-29119_reviewer.pdf [file pone.0258715.s003.pdf]

PLOS ONE

A local-ingredients-based supplement is an alternative to corn-soy blends plus for treating moderate acute malnutrition among children aged 6 to 59 months: A randomized controlled non-inferiority trial in Wolaita, Southern Ethiopia

--Manuscript Draft--

**Manuscript Number:**	PONE-D-20-29119
**Article Type:**	Clinical Trial
**Full Title:**	A local-ingredients-based supplement is an alternative to corn-soy blends plus for treating moderate acute malnutrition among children aged 6 to 59 months: A randomized controlled non-inferiority trial in Wolaita, Southern Ethiopia
**Short Title:**	Treating moderate acute malnutrition with local ingredient-based supplement in Southern Ethiopia.
**Corresponding Author:**	Debritu Nane, MSc. in human nutrition<br>Hawassa University and University of Bergen<br>Sodo, ETHIOPIA
**Keywords:**	Moderate acute malnutrition, local-ingredients-based supplement, corn-soya blends plus, randomized controlled non-inferiority trial
**Abstract:**	Globally, moderate acute malnutrition (MAM) affects approximately 5% of children below five years of age. In Ethiopia, 12.5% of children below five years are wasted, and 9.7% are moderately wasted. MAM is a persistent public health problem in Ethiopia. The current approach in Ethiopia for the management of MAM is a supplementary feeding program; however, this is only provided to chronically food-insecure areas. The objective of the study was to compare a local-ingredients-based supplement (LIBS) with the standard corn-soy blend plus (CSB+) in treating MAM among children aged 6 to 59 months to test the hypothesis that the recovery rate achieved with LIBS will not be more than 7% worse than that achieved with CSB+.<br>Methods and findings<br>We used an individual randomized controlled non-inferiority trial design with two arms, involving 324 children with MAM aged 6 to 59 months in Wolaita, Southern Ethiopia. One hundred and sixty-two children were randomly assigned to each of the two arms. In the first arm, 100 g of LIBS plus 25.2 g of sugar with 8 ml of vegetable oil/day was provided. In the second arm, 150 g of CSB+ with 16 ml of oil/day was provided. Both intention-to-treat (ITT) and per-protocol (PP) analyses were done.<br>ITT and PP analyses showed non-inferiority of LIBS compared with CSB+ for recovery rate [ITT risk difference = 3.7% (95% CI: 5.9, 13.2); PP risk difference = 4% (95% CI: –5.8, 13.7); average weight gain [ITT risk difference = 0.1 g (95% CI: -0.4 g, 0.6 g); PP risk difference = 0.14 g (95% CI: -0.3 g, 0.6 g)]; and recovery time [risk difference = -2.7 days (95% CI: 8.5 days, 3.1 days)]. There was no difference over 12 weeks in daily length/height gain and MUAC gain between LIBS and CSB+ groups (p=0.14).<br>Conclusions<br>LIBS is an effective alternative to the standard CSB+ for the treatment of MAM. Thus, the potential of scaling up the use of LIBS should be promoted.
**Order of Authors:**	Debritu Nane, MSc. in human nutrition
	Anne Hatløy
	Bernt Lindtjørn
**Additional Information:**	
**Question**	**Response**
**Financial Disclosure**<br><br>Enter a financial disclosure statement that describes the sources of funding for the work included in this submission. Review	The author who received the award of this study is Debritu Nane (corresponding author). The grant number of the award is ETH-13/0025. The funding organization of this research is NORAD (Norwegian Agency for Development Cooperation); through the NORHED program (Norwegian Program for Capacity Development in Higher Education and Research for Development). URL of the funder is “South Ethiopia

Powered by Editorial Manager® and ProduXion Manager® from Aries Systems Corporation

the submission guidelines for detailed requirements. View published research articles from *PLOS ONE* for specific examples.<br><br>This statement is required for submission and **will appear in the published article** if the submission is accepted. Please make sure it is accurate.<br><br>**Unfunded studies**<br>Enter: *The author(s) received no specific funding for this work.*<br><br>**Funded studies**<br>Enter a statement with the following details:<br>• Initials of the authors who received each award<br>• Grant numbers awarded to each author<br>• The full name of each funder<br>• URL of each funder website<br>• Did the sponsors or funders play any role in the study design, data collection and analysis, decision to publish, or preparation of the manuscript?<br>• **NO** - Include this sentence at the end of your statement: *The funders had no role in study design, data collection and analysis, decision to publish, or preparation of the manuscript.*<br>• **YES** - Specify the role(s) played.<br><br>* typeset	Network of Universities in Public Health (SENUPH) improving women's participation in post graduate education". This funding organization is responsible in the process of designing the study and data collection, analysis, and interpretation and in writing the manuscript.
**Competing Interests**<br><br>Use the instructions below to enter a competing interest statement for this submission. On behalf of all authors, disclose any competing interests that could be perceived to bias this work—acknowledging all financial support and any other relevant financial or non-financial competing interests.<br><br>This statement **will appear in the published article** if the submission is accepted. Please make sure it is accurate. View published research articles from *PLOS ONE* for specific examples.	The authors have declared that no competing interests exist.

Powered by Editorial Manager® and ProduXion Manager® from Aries Systems Corporation

**NO authors have competing interests**<br><br>Enter: *The authors have declared that no competing interests exist.*<br><br>**Authors with competing interests**<br><br>Enter competing interest details beginning with this statement:<br><br>*I have read the journal's policy and the authors of this manuscript have the following competing interests: [insert competing interests here]*<br><br>* typeset	
**Ethics Statement**<br><br>Enter an ethics statement for this submission. This statement is required if the study involved:<br><br>• Human participants<br>• Human specimens or tissue<br>• Vertebrate animals or cephalopods<br>• Vertebrate embryos or tissues<br>• Field research<br><br>Write "N/A" if the submission does not require an ethics statement.<br><br>General guidance is provided below. Consult the submission guidelines for detailed instructions. **Make sure that all information entered here is included in the Methods section of the manuscript.**	This study was approved by the Hawassa University College of Medicine and Health Sciences Institutional Review Board (IRB/024/10) and regional committees for medical and health research ethics in Norway (2018/69/REK vest). Written informed consent was obtained from all caregivers of children who met enrollment criteria before the recruitment of their children into the study.

Powered by Editorial Manager® and ProduXion Manager® from Aries Systems Corporation

**Format for specific study types**<br><br>**Human Subject Research (involving human participants and/or tissue)**<br>• Give the name of the institutional review board or ethics committee that approved the study<br>• Include the approval number and/or a statement indicating approval of this research<br>• Indicate the form of consent obtained (written/oral) or the reason that consent was not obtained (e.g. the data were analyzed anonymously)<br><br>**Animal Research (involving vertebrate animals, embryos or tissues)**<br>• Provide the name of the Institutional Animal Care and Use Committee (IACUC) or other relevant ethics board that reviewed the study protocol, and indicate whether they approved this research or granted a formal waiver of ethical approval<br>• Include an approval number if one was obtained<br>• If the study involved *non-human primates*, add *additional details* about animal welfare and steps taken to ameliorate suffering<br>• If anesthesia, euthanasia, or any kind of animal sacrifice is part of the study, include briefly which substances and/or methods were applied<br><br>**Field Research**<br><br>Include the following details if this study involves the collection of plant, animal, or other materials from a natural setting:<br>• Field permit number<br>• Name of the institution or relevant body that granted permission	
**Data Availability**<br><br>Authors are required to make all data underlying the findings described fully available, without restriction, and from the time of publication. PLOS allows rare exceptions to address legal and ethical concerns. See the PLOS Data Policy and FAQ for detailed information.	No - some restrictions will apply

Powered by Editorial Manager® and ProduXion Manager® from Aries Systems Corporation

A Data Availability Statement describing where the data can be found is required at submission. Your answers to this question constitute the Data Availability Statement and **will be published in the article**, if accepted.<br><br>**Important:** Stating 'data available on request from the author' is not sufficient. If your data are only available upon request, select 'No' for the first question and explain your exceptional situation in the text box.<br><br>Do the authors confirm that all data underlying the findings described in their manuscript are fully available without restriction?	
**Describe where the data may be found in full sentences. If you are copying our sample text, replace any instances of XXX with the appropriate details.**<br><br>• If the data are **held or will be held in a public repository**, include URLs, accession numbers or DOIs. If this information will only be available after acceptance, indicate this by ticking the box below. For example: *All XXX files are available from the XXX database (accession number(s) XXX, XXX.).*<br>• If the data are all contained **within the manuscript and/or Supporting Information files**, enter the following: *All relevant data are within the manuscript and its Supporting Information files.*<br>• If neither of these applies but you are able to provide **details of access elsewhere**, with or without limitations, please do so. For example:<br><br>*Data cannot be shared publicly because of [XXX]. Data are available from the XXX Institutional Data Access / Ethics Committee (contact via XXX) for researchers who meet the criteria for access to confidential data.*<br><br>*The data underlying the results presented in the study are available from (include the name of the third party*	The datasets used and/or analyzed during the current study will be available from the corresponding author, on reasonable request.

and contact information or URL).<br>• This text is appropriate if the data are owned by a third party and authors do not have permission to share the data.<br><br>* typeset	
Additional data availability information:	

Powered by Editorial Manager® and ProduXion Manager® from Aries Systems Corporation

Manuscript

Click here to access/download;Manuscript;Debritu_manuscript3plos.DOCX

A local-ingredients-based supplement is an alternative to corn-soy blends plus for treating moderate acute malnutrition among children aged 6 to 59 months: A randomized controlled non-inferiority trial in Wolaita, Southern Ethiopia

Debritu Nane[1,2*], Anne Hatløy[2,3], Bernt Lindtjorn[1,2]

1. Hawassa University, School of Public and Environmental Health, PO Box 5, Ethiopia.
2. University of Bergen, Centre for International Health, PO Box 7800, 5020 Bergen, Norway
3. Fafo Institute for Labour and Social Research, PO Box 2947, 0608 Oslo, Norway

[2,3]anne.hatloy@uib.no

[2]bernt.lindtjorn@cih.uib.no

***Corresponding author:** nanedebritu@gmail.com

Abstract

Background

Globally, moderate acute malnutrition (MAM) affects approximately 5% of children below five years of age. In Ethiopia, 12.5% of children below five years are wasted, and 9.7%

define wasted scientifically, and / or replace in abstrct with something like malnourished

are moderately wasted. MAM is a persistent public health problem in Ethiopia. The current approach in Ethiopia for the management of MAM is a supplementary feeding program; however, this is only provided to chronically food-insecure areas. The objective of the study was to compare a local-ingredients-based supplement (LIBS) with the standard corn-soy blend plus (CSB+) in treating MAM among children aged 6 to 59 months to test the hypothesis that the recovery rate achieved with LIBS will not be more than 7% worse than that achieved with CSB+.

Methods and findings

We used an individual randomized controlled non-inferiority trial design with two arms, involving 324 children with MAM aged 6 to 59 months in Wolaita, Southern Ethiopia. One hundred and sixty-two children were randomly assigned to each of the two arms. In the first arm,

sucrose! toxic specify

100 g of LIBS plus 25.2 g of sugar with 8 ml of vegetable oil/day was provided. In the second

specify oil

arm, 150 g of CSB+ with 16 ml of oil/day was provided. Both intention-to-treat (ITT) and per-protocol (PP) analyses were done.

ITT and PP analyses showed non-inferiority of LIBS compared with CSB+ for recovery rate [ITT risk difference = 3.7% (95% CI: 5.9, 13.2); PP risk difference = 4% (95% CI: –5.8, 13.7); average weight gain [ITT risk difference = 0.1 g (95% CI: -0.4 g, 0.6 g); PP risk difference = 0.14 g (95% CI: -0.3 g, 0.6 g)]; and recovery time [risk difference = -2.7 days (95% CI: 8.5 days,

3.1 days)]. There was no difference over 12 weeks in daily length/height gain and MUAC gain between LIBS and CSB+ groups (p=0.14).

Conclusions

LIBS is an effective alternative to the standard CSB+ for the treatment of MAM. Thus, the potential of scaling up the use of LIBS should be promoted.

Trial registration: Pan-African Clinical Trial Registration number: PACTR201809662822990.

Keywords: Moderate acute malnutrition, local-ingredients-based supplement, corn-soya blends plus, randomized controlled non-inferiority trial

Introduction

Globally, acute malnutrition remains a major public health problem, affecting an estimated 55 million children below five years of age [1, 2]. Acute malnutrition is possibly caused by prolonged food insecurity, poverty, poor feeding practices, inadequate household food accessibility, and infections [3, 4]. Weakness of immune function, decreased desire for food, increased metabolic rate and nutrient requirements, and increased susceptibility to infection and illness are considered to be potential outcomes of acute malnutrition [5].

Acute malnutrition is categorized as moderate or severe [6]. Moderate acute malnutrition

explain what aspect of impaired growth and development is "acute". Malnutrition must be chronic and / or frequently repeated for impaired growth and development

(MAM), defined by weight-for-height z-score (WHZ) between -3 and -2 standard deviations and/or mid-upper arm circumference (MUAC) of between ≥11.5 cm and <12.5 cm, is one form of acute malnutrition [7-9]. Severe acute malnutrition (SAM) is defined as WHZ <-3 SD or a MUAC of <110 mm, or the presence of nutritional edema [6, 10]. Acute malnutrition is implicated in approximately 14.6% of deaths occurring among wasted children aged below five years. Of this, 10.2% is due to MAM, and 4.4% is due to SAM [11].

According to the Ethiopian Demographic Health Survey (EDHS) 2011, about 10% of

acute means what in terms of nutrient delivery????

children aged below five years of age suffered from acute malnutrition, and among these, 70% had MAM [12]. Children with MAM have a three-fold higher risk of morbidity and mortality than their normal counterparts [1, 11].

Even though MAM is more prevalent than SAM, and MAM can progress to a life-threatening condition [9, 13], not much information is available on the management of MAM [14, 15]. Untreated, children suffering from MAM can stay moderately wasted for several months, probably worsening future linear growth and cognitive development [16]. Thus, the management of MAM should be considered a public health priority [9].

There is presently no standardized approach to managing MAM, especially in non-emergency and relatively food secure settings of low- and middle-income countries [8, 16, 17]. In Ethiopia, the current approach for the management of MAM is a supplementary feeding program (SFP), although this is limited to areas where chronic food insecurity exists. In districts not determined to be persistently food insecure, there are no food supplementation strategies for MAM children. In these districts, the approach to managing children suffering from MAM includes vitamin A supplementation, deworming, and dietary counseling delivered to the caregivers of children [12, 18]. The nutritional counseling is delivered to the caregivers of children, assuming that they have access to all foods needed for child feeding, but lack information on how best to use them [19]. However, studies show that the recovery rate from MAM is unsatisfactory among children aged 6 to 59 months who get health and dietary counseling services alone [18].

The dietary supplementation of children with MAM enhances their recovery from MAM, with a reduced risk of morbidity [20]. Children with MAM are commonly treated with fortified blended flours, especially corn-soy blend (CSB), super cereal/corn-soy blend plus (CSB+), and super cereal plus/corn-soy blend plus with milk and oil (CSB++) [8, 11, 21]. The current food blends used for the management of MAM are CSB+ and CSB++. CSB+ contains maize (64%), whole soya beans (24%), sugar (10%), vegetable oil, and vitamin and mineral premix. CSB++ is made from maize (58%), dehulled soya beans (20%), dried skimmed milk powder (8%), sugar (10%), vegetable oil, and vitamin and mineral premix; it is used as a complement to breast milk. These cereal and soya bean combinations have been refined, blended, and precooked (by roasting) for children with MAM below five years of age [8]. These standard food supplements

specify

specify if sucrose or what

specify

what cereals and grains??

are effective in treating MAM; however, such management is temporary and not sustainable for the management of recurrent malnutrition in poverty-hit countries [11].

The dietary management of MAM could be done by using locally accessible nutrient-dense foods [9]. Local ingredients that are available and accessible can be formulated at the household level; they are easy to formulate, cheap, and yet they contain adequate amounts of the required nutrients [11]. In this paper, the new supplementary food is referred to as a local-ingredients-based supplement (LIBS); it is made of locally available ingredients such as pumpkin seed, peanut, amaranth grain, flaxseed, and emmer wheat. The proportions of these ingredients were designed to have the required amount of nutrients for the management of MAM among children aged 6 to 59 months [9].

According to a study done in Uganda, the recovery rates after supplementation with malted sorghum-based porridge versus CSB+ resulted in similar recovery rates among MAM children [11]. Another study showed no significant difference in recovery rate between CSB+ and ready-to-use supplementary foods (RUSF), which were relatively successful for the treatment of MAM [7]. The recovery rates were also not significantly different in a study from Myanmar for a sorghum-peanut blend group and a CSB+ group among children with MAM [18]. Since CSB+ (the standard treatment) had similar effects compared to other food supplements prepared for the treatment of MAM, the differences are too narrow to feasibly conduct a superiority trial. We, therefore, conducted a non-inferiority trial to demonstrate if LIBS is at least as effective as CSB+ in treating MAM.

To resolve the limited delivery of supplements among children with MAM, the usefulness of LIBS needs to be explored. Besides this, adequate research has not been conducted in Ethiopia on LIBS, even though it has the potential to contribute to treating MAM. The

evidence drawn from this study will be shared with the public, policymakers, and scholars, as LIBS has the potential to increase recovery from MAM and decrease the burden of malnutrition. This study aims to evaluate if LIBS is as effective as standard corn-soy blend plus (CSB+) in treating MAM among children aged 6 to 59 months.

Introduction needs better focus; reduce length by at least 30%; restrict to introduction, not discussion

Materials and methods

require an ethics statement

Subject and setting

We conducted the study in Damot Pulassa district in Wolaita zone in the South-Western part of Ethiopia, where there is a high level of food insecurity and child malnutrition. Damot Pulassa is characterized by having a fragmented farm and land ownership. Damot Pulassa has the highest population density in Ethiopia reaching up to 600 persons per square kilometer in some *Kebeles* (small villages) of it. The discrepancy between population and land balance has by far continued to be the primary cause for the endemic food insecurity in the area [22]. According to a study protocol of this trial, Damot Pulassa district is characterized as a maize and root crop livelihood area as these are the main crops cultivated in the district [3]. This district has five health centers and 23 health posts that are led by health extension workers. These health facilities deliver nutrition-linked services like counseling on feeding practices, screening for nutritional status of young children, and nutritional management of the acutely malnourished children.

Children aged 6 to 59 months with MAM, living in selected *kebeles* of Damot Pulassa district were included in the study. Children were excluded if they had SAM (based on WHO 2009 child growth standards), and/or if they had bilateral pitting edema [23], or had any illness or other medical complications that prevented the children from safely consuming supplementary food. Children were also excluded if they were simultaneously involved in another

supplementary feeding program. Recruited children were identified as MAM according to their MUAC values, and not only according to the values of weight-for-height/length z-scores. The respondents were mothers or caregivers of selected children.

Study design and intervention

This was a randomized, controlled, non-inferiority trial that assessed the efficacy of 100 g of LIBS with 20 g of sugar plus 8 ml of oil/day (the intervention), compared with conventional treatment, which is CSB+ in the amount of 150 g flour/day with 16 ml of oil (the control), in treating MAM for a period of 12 weeks.

Objective

The objective of this study was to test if children aged 6 to 59 months suffering from MAM, treated with 100 g of LIBS, 20 g of sugar and 8 ml of oil/day, for a period of 12 weeks, would not have an inferior recovery rate compared to similar children treated with 150 g CSB+ flour and 16 ml of oil/day (with a 7% margin of non-inferiority).

Outcome variables and quality measurements

The primary outcome was recovery rate: percentage of children who attained a MUAC ≥12.5 cm and/or WHZ ≥ -2 without bipedal edema at the end of 12 weeks. The secondary outcomes were the mean recovery time (the duration within 12 weeks in which the child recovered from MAM) and average weight gain. Children who progressed to SAM during the study and/or persisted as moderately malnourished at the end of the 12-week follow-up were considered to have failed management for MAM. The selection of outcome measures was based on similar studies [11, 24, 25].

The two research teams included two supervisors, 12 data collectors and 18 food distributers. The supervisors and data collectors were trained by the principal investigator for

seven days before commencement of the study. The training covered the objectives of the study, data collection systems, questions found in the questionnaire, interview techniques, and anthropometric measurements. Training in anthropometric measurement techniques, periodic standardization, and daily calibration of equipment was done based on WHO recommendations for anthropometric measurement protocols [26]. The food distributers were trained for five days on supplement distribution technique, feeding procedures, and cooking of the supplementary foods before study commencement.

When the child was a twin, we provided an additional amount of supplementary food to the caregiver to ensure that the enrolled child was fed with a full portion. When two children with MAM were found in the same household, we provided similar supplements for both children but only the randomly selected child was taken as a study subject. When the recruited child was not at home during the time of follow-up, data collectors revisited such households until they found the child. Supervisors oversaw activities daily. The food distribution process, feeding techniques, and use of the provided food supplements were monitored among randomly selected households on a twice-monthly basis.

Sample size calculation

We calculated the sample size to investigate if LIBS was not inferior to CSB+ in terms of the recovery rate among children with MAM aged 6 to 59 months. The sample size was calculated based on 80% power of the test, 7% margin of non-inferiority, 5% level of significance, and assuming a recovery rate with CSB+ of 67%. The planned sample size for the study was 162 subjects per study group, allowing for a 10 percent withdrawal rate. This led to a total required sample size of 324 subjects. The anticipated 10 percent dropout rate was used based on the observed dropout rate reported in two studies [11, 24]. The non-inferiority margin

was set depending on the reported recovery rates from MAM [21, 24]. For this sample size calculation, the PharmaSchool sample size calculator for non-inferiority trials was used.

Recruitment of study participants

Skilled data collectors, accompanied by health extension workers, visited all households found in randomly selected *kebeles* in Damot Pulassa district with children aged 6 to 59 months, to assess children for eligibility by measuring MUAC. The recruitment was done from August 27 to September 20, 2018. Children with MUAC <12.5 cm were recorded and brought to the actual screening site with their mother/caregiver, where MUAC was re-measured, and weight and height or length were taken. In addition to the values of MUAC (i.e. between ≥11.5 cm and <12.5 cm), we used the WHZ or weight for length z-score for recruitment (i.e. WHZ: between −3 and −2 Z-scores). When the child was identified as moderately malnourished according to the MUAC and not according to the values of weight-for-height/length z-scores, we recruited them according to their MUAC values. Children aged 6 to 23 months were measured for weight and length, and children aged 24 to 59 months were measured for weight and height. Edematous malnutrition was also assessed using the bilateral pitting edema criterion. The screening for MAM was continued until the sample size was met.

Randomization

A computer-generated, sequentially numbered randomization list that contained codes for children who met enrolment criteria was prepared by one of the research team, who was not otherwise involved with the study. Allocation into study groups was done using random allocation software. The allocation ratio was 1:1, and the children were recruited into groups according to the randomization list. One of the research team, who had no information about the subjects' identity, allocated the participants to the intervention/control groups. These children

were further classified by the investigator into sub-groups, with equal numbers of subjects based on their neighborhood. This was done to facilitate assigning one food distributor per sub-group, where food distributors simply had to access the households with a selected child.

Blinding

This study was double-blinded, that is, caregivers, data collectors, and food distributors were blinded for the intervention. Both supplements were packed with the same plastic packs, similar in color and texture, and they were prepared and distributed in the same way. The sugar which was provided for the intervention group was blended with the flour before packing. Vegetable oil was distributed with the supplement for both intervention and control groups. Food distributors were assigned to support caregivers in cooking the porridge with 8 ml of oil for the intervention group and with 16 ml of oil for the CSB+ group. We distributed the oil with colored plastic cups. Data were analyzed by an individual other than the assessor.

Data collection and follow-up

Before introducing the interviews, the study questionnaires were subjected to pilot-testing and were refined for clarity and correctness. The interviews were conducted with selected caregivers of children with MAM aged 6 to 59 months, for collection of baseline information such as socio-demographic and economic status, child's age, dietary habits, breastfeeding practices, and history of child and maternal illness. The child's weight was measured with a Seca weight scale to the nearest 0.1 kg. The data collectors ensured that the scale was positioned on a flat, firm surface, and weighing was done with light clothing.

The length was measured to the nearest 0.1 cm, using a locally prepared wooden measuring board for children aged 6 to 23 months. For children aged 24 to 59 months, height was measured to the nearest 0.1 cm using a Seca height scale. Before height measurement, the

data collectors ensured that the height board was on level ground and the child was barefoot; the collector kneeled to get to the level of the child and encouraged the caregiver to help. For length, data collectors measured the child lying down, being sure that the length board was placed on a flat and stable surface.

MUAC was assessed by non-stretchable standard United Nations Children's Fund (UNICEF) plastic tape measures. The measurement was taken half-way between the acromion and the olecranon processes, with the measuring tape fitting comfortably, but without making a depression on the left upper arm. This was done twice for every child, using two different data collectors, and the average of the two measures was recorded to the nearest 0.1 cm. Participants in both groups were visited every week for consecutive 12weeks to collect anthropometric measurements using MUAC, and information on supplement use and morbidity. Every month, anthropometric data were collected with identical equipment as used at baseline. Bilateral pitting edema was assessed by pressing for three seconds on the dorsum of the foot. Based on the follow-up measurement of anthropometry, children who developed SAM were sent to the SAM clinic.

Description of interventions and distribution of supplementary foods

Subjects in the intervention group received a daily ration of 100 g of LIBS with 20 g of sugar for 12 weeks. The composition of LIBS was: 30 g of pumpkin seed, 25 g of peanut grain, 20 g of amaranth grain, 15 g of flaxseed, 10 g of emmer wheat, 25.2 g of sugar and 8 ml of oil.

provide details - specifics

This supplement yielded 699 kcal, 22.6 g protein, [and g of carbs?] and 40.89 g fat. Likewise, children in the control group received CSB+ in the amount of 150 g flour [specify]/day with 16 ml of oil [specify]; this yielded 764 kcal, 20.25 g protein, [carbs??] and 30.76 g fat daily for 12 weeks. The participants from both groups were served with the supplements in the morning as breakfast (Table 1). Children aged 6–23 months

and 24 to 59 months received a similar amount of food [26]. The food distributors who were trained for the preparation of porridge visited each household daily to help the caregivers in the preparation of the porridge and feeding, to advise, assess, and resolve problems with feeding. Also, they checked for and measured the amount of supplement consumed by the children.

Table 1. Nutrient composition of the supplementary foods.

Nutrient	100 gm of LIBS with 25.2 gm of sugar plus 8 ml oil	150 gm of CSB+ with 16ml of oil
**Energy (kcl)**	698.5	763.98
**Protein (gm)**	22.6	20.25
**Fat (gm)**	40.89	30.76
**Iron (mg)**	8.1	6.00
**Zn (mg)**	5.6	7.5
**Calcium (mg)**	100.00	195.00
**Phosphorous (mg)**	470.55	300.00
**Potassium (mg)**	666.14	600.00
**Magnesium (mg)**	394.7	107.75
**Sodium (mg)**	84.6	41.25
**Folic acid (µg)**	49.4	90.00

carbs?
ash?

Abbreviations: LIBS: local-ingredients-based supplement; CSB+: corn-soy blend plus; kcal: kilo calorie; g: gram; and mg: milligram.

Note: Nutrient values for the LIBS ration were calculated by using the United States Department of Agriculture (USDA) food composition database and NutriSurvey software. Nutrient values for the CSB+ were adapted from Amegovu KA, Ogwok P, Ochola S, Yiga P, Musalima HJ, Mutenyo E. Formulation of sorghum-peanut blend using linear programming for treatment of moderate acute malnutrition in Uganda. J Food Chem and Nutr. 2013; 1(2):67–77.

Data analysis

All data collection sheets were controlled by the field supervisors for completeness. Data entry was done using Epi Data version 3.1. Double data entry was done simultaneously to ensure data quality. According to the recommendations for analyzing and reporting equivalence and non-inferiority trials, both intention-to-treat (ITT) and per-protocol (PP) analyses were done, and the 95 percent confidence interval (CI) level was used to interpret any differences [24]. The ITT analyses involved all patients who were randomly allocated, whereas the PP analysis excluded children who refused, transferred out of the management program, or were lost to follow-up, but included children who died, or were discharged as cured or non-cured. Statistical analysis was done using SPSS and STATA software.

Comparisons of baseline and outcomes characteristics between CSB+ and LIBS were made by using Fisher's exact test, chi-square test for dichotomous variables, and Student's t-test for continuous variables. Means were compared with the use of t-test, medians with the use of the Mantel-Haenszel test, and proportions with the use of Student's chi-square test. Differences in the estimated proportion between the groups along with 95 percent CI were estimated to infer non-inferiority. The recovery time between groups was predicted in Kaplan-Meier (log-rank) curves of survival analysis. P values <0.05 were considered to be statistically significant. Mean difference of recovery time, mean difference between weight gains, mean difference of MUAC gain, and mean difference of height/length gain were computed to describe the magnitude of the difference between the two groups. Rates of weight gain through the whole period of follow-up were estimated in g/kg body weight/day and compared between the study groups. These were calculated by dividing the weight gain (weight at exit minus weight at admission), expressed in

grams, by the weight at admission (in kilograms) and the length of stay (in days). Anthropometric indexes were calculated using ENA for SMART 2011 software.

Ethics approval and consent to participate

needs to be at the beginning of the Methods section

This study was approved by the Hawassa University College of Medicine and Health Sciences Institutional Review Board (IRB/024/10) and regional committees for medical and health research ethics in Norway (2018/69/REK vest).The approval obtained from Hawassa University College of Medicine and Health Sciences institutional review board covered all sites included in the study. The purpose of the study and methods of data collection, confidentiality, and voluntary participation were explained to mothers of children who were invited to sign an informed consent form. Written informed consent was obtained from all caregivers of children who met enrollment criteria before the recruitment of their children into the study. All interviews and intervention procedures were conducted in privacy. No serious adverse reactions were detected.

Results

Enrollment process and trial profile for the study participants

A total of 1006 children were screened for MAM, of whom 682 children were excluded because 650 children were without MAM and 32 children did not meet inclusion criteria. In the study, 324 children with MAM aged 6 to 59 months were enrolled to either LIBS (*n*=162) or CSB+ (*n*=162). Of these, 311 (96%) children with MAM and their mothers/caregivers completed the study, with seven lost to follow-up and six discontinued due to SAM. The reason for all those lost to follow-ups was moving from the study site. The enrollment and allocation of participants, lost to follow-ups, discontinued, and completed the study are illustrated in **(Fig 1).**

Fig 1. Flowchart of study enrolment to completion.

(A) Screened for MAM, children aged 6 to 59 months who screened for moderate acute malnutrition. (B) Children without MAM, children aged 6 to 59 months who did not have moderate acute malnutrition. (C) Randomized children aged 6 to 59 months who assessed positive for moderate acute malnutrition, fulfilled the inclusion criteria, were recruited and randomly allocated. (D) Loss to follow-up, children who were randomly allocated to intervention and control groups and stopped participating in the study at any stage of the study. (E) Discontinued due to SAM, children who were randomly allocated to intervention and control groups and discontinued from the study at any stage of the study. (F) Analyzed at 12 weeks, children aged 6 to 59 months whose information was analyzed at 12 weeks. (G) Abbreviations: MAM: moderate acute malnutrition; LIBS: local-ingredients-based supplement; CSB+: corn-soy blend plus; SAM: severe acute malnutrition.

Baseline characteristics of the study participants

Baseline characteristics were compared between the LIBS and CSB+ groups (Table 2). No significant differences ($p<0·05$) were noted between LIBS and CSB+ groups. Most of the mothers/caregivers were within the age range of 18 to 30 years. The mean (SD) age of the children in the LIBS group was 20.1 (3.5) months and 18.5 (3.6) months in the CSB+ group. The majority of the children were from agricultural households, with six to eight persons per household and with a low rate of literacy. More than half of children, 54.9% of children in the LIBS group, and 64.2% of children in the CSB+ group, had morbidity history based on two weeks recall.

Table 2. Baseline characteristics of study subjects per treatment and control group.

Characteristics (N=324)	LIBS (n=162)	CSB+ (n=162)	P-value
**Sex of child [n (%)]**			
Male	67 (41.4)	70 (43.2)	0.74
Female	95 (58.6)	92 (56.8)	
**Age of the child in months [n (%)]**			
6 to 24	106 (65.4)	112 (69.1)	0.4
>24	56 (34.6)	50 (30.9)	
**Age of respondents in years [n (%)]**			
18 to 30	98 (60.5)	82 (50.60	0.2
31 to 40	60 (37)	75 (46.3)	
>40	4 (2.5)	5 (3.1)	
**The education level of respondents [n (%)]**			
Non-formal education	111 (68.5)	112 (69.1)	0.1
Primary	46 (28.4)	37 (22.8)	
Secondary and above	5 (3.1)	13 (8.1)	
**Occupation of the household head [n (%)]**			
Farmer	94 (58)	74 (45.7)	0.14
Daily worker	26 (16.1)	40 (24.7)	
Business owner	30 (18.5)	33 (20.4)	
Monthly paid worker	6 (3.7)	8 (4.9)	
Handcrafts	6 (3.7)	7 (4.3)	
**Morbidity based on two weeks recall [n (%)]**			
Yes	89 (54.9)	104 (64.2)	0.09
No	73 (45.1)	58 (35.8)	
**Household size [n (%)]**			
3 to 5	42 (25.9)	47 (29)	0.1
6 to 8	96 (59.3)	103 (63.6)	
>8	24 (14.8)	12 (7.4)	
**Use of bed net by the child [n (%)]**			
Yes	128 (79)	116 (71.6)	0.1
No	34 (21)	46 (28.4)	
**MUAC of the child (cm), mean (SD)**	12.01 (1.03)	12.05 (1.03)	0.3
**Weight of the child (kg), mean (SD)**	8 (1.2)	7.9 (1.2)	0.4
**Height of the child (cm), mean (SD)**	76.6 (1.1)	76.3 (1.2)	0.75
**WHZ, mean (SD)**	-2.3 (0.2)	-2.3 (0.23)	0.46

LIBS: local-ingredients-based supplement; CSB+: improved corn-soya blend; WHZ: weight-for-age Z score; MUAC: mid-upper arm circumference.

Outcomes in the two intervention arms over 12 weeks

The overall proportion of children who recovered was 72.5%, whereas 1.9% deteriorated to SAM, 23.5% remained in MAM, and 2.2% were lost to follow-ups at the end of the 12-week intervention. The proportion of children who recovered was similar for LIBS and CSB+ groups in both ITT and PP analysis. The ITT analysis showed recovery of 75.3% for LIBS (95% CI: 68.1, 81.3) and 71.6% for CSB+ (95% CI: 64.2, 78.0) (p=0.5), whereas the PP analysis showed recovery of 73.5 percent for LIBS (95% CI: 66.0, 80.0) and 70.4% for CSB+ (95% CI: 63, 77) (p=0.8) (Table 3). The risk differences for LIBS compared with CSB+ were 3.7% (95% CI: -5.9, 13.2) in ITT analysis and risk difference = 4.0% (95% CI: -5.8, 13.7) in PP analysis. Both PP and ITT analyses showed that LIBS was non-inferior compared with CSB+ in terms of the recovery rate, as the lower limit of 95% CI of the difference between the two groups was above the predefined non-inferiority margin of risk difference (-7%) **(Fig 2)**.

Fig 2. Difference in recovery rate between LIBS and CSB+ groups.

(A) The recovery rate in the LIBS group was non-inferior compared with the CSB+ group. (B) Numbers are risk differences of recovery rates between groups (%). (C) Dotted line indicates the predefined non-inferiority margin. (D) Abbreviations: LIBS: local-ingredients-based supplement; CSB+: corn-soy blend plus; PP: per-protocol; ITT: intention to treat.

Table 3. Outcomes in the two intervention arms over 12 weeks.

Outcomes	All (N=324)	LIBS (n=162)	CSB+ (n=162)	P-value
**Recovery [n (%)]**	238 (73.5)	122 (75.3)	116 (71.6)	0.8
**SAM [n (%)]**	6 (1.9)	2 (1.2)	4 (2.5)	0.8
**MAM [n (%)]**	78 (24.1)	38 (23.5)	40 (24.7)	0.8
**Loss to follow-up [n (%)]**	7 (2.2)	3 (2.5)	4 (2.5)	0.8
**Weight gain (g/kg/day ±SD)**	3.2 (2.1)	3.2 (2.1)	3.1 (2.1)	0.4
**MUAC gain (mm/day ±SD)**	0.12 (0.08)	0.12 (0.07)	0.12 (0.08)	0.2
**Height gain (mm/day ±SD)**	0.4 (0.3)	0.4 (0.02)	0.35 (0.02)	0.2

LIBS: local-ingredients-based supplement; CSB+: improved corn-soya blend; g: gram; kg: kilogram; MUAC: mid-upper arm circumference; mm: millimeter

The daily weight gain was similar in both groups. The mean weight gain (SD) among children in the LIBS group was 3.2 (2.1) g/kg/day, whereas the mean weight gain (SD) among children in the CSB+ group was 3.1 (2.1) g/kg/day (p=0.4) (Table 3). Both ITT and PP analyses showed that the LIBS group was non-inferior compared with the CSB+ group (predefined non-inferiority margin of risk difference = -1.3 g/kg/d) [ITT risk difference = 0.1 (95% CI: -0.4, 0.6); PP risk difference = 0.14 (95% CI: -0.3, 0.6)] **(Fig 3)**. The mean MUAC gain (SD) for the LIBS group was 0.12 (0.07) mm/day and 0.11 (0.08) mm/day for the CSB+ group (p=0.2). There was no difference in daily length/height gain over 12 weeks between the LIBS and CSB+ groups (p=0.14) (Table 3).

Fig 3. Difference in average weight gain (g/kg/day) between LIBS and CSB+ groups.

(A) The average daily weight gain in the LIBS group was non-inferior compared with the CSB+ group. (B) Numbers are risk differences of average weight gains between groups (g/kg/day). (C) Dotted line indicates the predefined non-inferiority margin. (D) Abbreviations: LIBS: local-

ingredients-based supplement; CSB+: corn-soy blend plus; PP: per-protocol; ITT: intention-to-treat.

Overall, the mean recovery time (predefined non-inferiority margin of risk difference = 14 days) in the ITT analysis for the LIBS group was 54.3 days (95% CI: 50, 58 days) and 57 days for those in the CSB+ group (95% CI: 53, 61 days), giving a difference of -2.7 days (95% CI: -8.5, 3.1 days). The difference in recovery time was not statistically significant (p=0.8) **(Fig 4)**.

Fig 4. Recovery of children with moderate acute malnutrition over time in the LIBS and CSB+ arms.

(A) Recovery of children with MAM over time in the LIBS group was similar to the CSB group (p=0.8). (B) Survival analysis, Kaplan-Meier curves. (C) Log-rank tests were used to analyze and describe the data. (D) Abbreviations: MAM: moderate acute malnutrition; LIBS: local-ingredients-based supplement; CSB+: corn-soy blend plus.

Discussion

This randomized controlled non-inferiority trial aimed to compare the efficacy of LIBS and CSB+ in treating MAM. In this study, the ITT as well as PP analyses showed that the children with MAM who received LIBS were not inferior compared with those who received CSB+ in terms of recovery rate, weight gain recovery time, MUAC gain, and length/height gain. Only the LIBS groups met the Sphere goal of 75% recovery.

The recovery rate of MAM observed in this study was comparable with the recovery rates of MAM with supplementary feeding reported in other studies (67% to 82.3%) [7, 11, 21, 27] and higher than the recovery rates of MAM with child-centered counseling intervention only [27]. The comparability of recovery rate with other studies, the non-inferiority of LIBS when

compared with CSB+, and the fact that the PP and ITT analyses presented similar results of recovery rate are strong evidence indicating that LIBS is an excellent alternative to CSB+ in treating MAM [28].

Additionally, these results are in line with findings of a study done in Uganda where standard therapeutic food for MAM (CSB+) and a locally produced supplement resulted in a comparable recovery rate among wasted children [11]. By contrast, this finding was inconsistent with a study done in Malawi [25], where the efficacy of CSB++ was compared with that of two RUSFs in treating MAM.

A possible explanation for the comparable recovery rate between LIBS and CSB+ groups could be related to both supplements having a similar energy density, as the energy density found in supplementary food is important for the recovery of MAM children [29]. During malnutrition, energy is needed to enhance catch-up growth and maintain the replacement of the loss of both lean and fat tissue [15].

In this study, the overall rate of loss to follow-up was similar between the LIBS and CSB+ groups and met the Sphere target rates for defaulting (≤15%). In the present study, the loss-to-follow-up rate was remarkably low (2.2%), much lower than that of previous studies, which had default rates of 4% to ≥5% [30-32]. This might be due to the weekly follow-up done by the researchers and the daily visits done by food distributors.

CSB+ has been used as a standard therapeutic food for MAM for a long time and is very well accepted, with a renowned feeding protocol in the study area. CSB+ is organoleptically tolerable in various settings [25]. Similarly, LIBS was developed from culturally known and acceptable foods and prepared with a similar appearance to the conventional food (CSB+). This could be another explanation for the comparability of the default rate between the two groups.

About 2% of children recruited in this study did not respond to treatment, but continued to lose weight and developed SAM. There was no significant difference in the number of children who developed SAM between the two groups. The reason for the progression to SAM is unknown and might not be related only to household food insecurity, in that it could be explained by the hypothesis that these children had an untreated illness. However, this rate of progression to SAM finding is lower than studies done in Cameroon [7], Malawi [25], and Burkina Faso [27].

This study showed that the average weight gain observed in the LIBS group was not inferior to the CSB+ group. This could be explained by the fact that the total energy provided by the supplements in both groups was comparable. Another explanation might be that both supplements need to be cooked before feeding to the child. The amount of water needed for cooking of both supplements was similar in that the mass of supplement fed to children was comparable. The finding also compared favorably with weight gain observed in a study carried out in Malawi [25] and was higher than studies done in Cameroon and Malawi [7, 33].

In the present study, the recovery time between the LIBS and CSB+ groups was similar. This finding is supported by other studies, for example, in Cameroon, where the efficacy of CSB+ and RUSF were compared in treating MAM [7], and in Uganda, where the efficacy of sorghum-peanut blend and CSB+ had been compared as therapeutic food for MAM [11]. By contrast, the recovery time was extended compared with a study done in Burkina Faso [27] However, it was within the acceptable Sphere Standards (≤ 90 days) [11].

The length/height gain was not comparable between the two groups. This could be explained by the similar amount of protein and zinc found in the LIBS and CSB+. Complementary, supplementary, and therapeutic foods with good quality protein have been

found to be effective for promoting the growth of children [34]. Similarly, zinc is vital for growth and development, and is a growth type II [15]. It has direct effects on the key hormonal system (IGF-I/GH) that controls growth in the postnatal phase [35]. Our finding was comparable with the study done by Stobaugh [36] and better than studies done by LaGrone et al., Nikièma et al. and Patel et al. [25, 27, 33].

This non-inferiority trial, to the best of our knowledge, was the first study so far using locally developed supplements based on local food ingredients to compare with the standard treatment of MAM (CSB+) in Ethiopia. One of the strengths of this study is that we have used locally available and culturally acceptable food ingredients to formulate the supplement. Another strength is meeting the Sphere Standard regarding recovery rate, recovery time, and loss to follow-up rate. The daily home visits by food distributors and weekly home follow-ups by the data collectors allowed us to minimize the loss-to-follow-up rate. However, the study had some limitations. The main limitation of this study was not monitoring the children's overall dietary intake during the treatment and not having a non-supplemented control group. Thus we could not measure the contribution of the home diet. Another limitation of this study was that we did not test the shelf life of the newly developed LIBS product. The shelf life of the product is critical if this product is to be used in the future as therapeutic food for treating children with MAM.

Conclusion

The present study showed that the LIBS was non-inferior to the conventional food (CSB+) in treating MAM, and both supplements were relatively successful for the treatment of MAM in children aged 6 to 59 months. The finding from this study provides an experimental indication that LIBS can be used as an alternative to CSB+ in the management of MAM in Ethiopia. Thus, the potential for scaling up the use of LIBS should be promoted. Further studies

should also examine concerns such as the product's shelf life, and its sustainability and cost-effectiveness. Community perceptions of the LIBS as a treatment for MAM should also be tested.

Acknowledgments

We thank all the participating children and their mothers or caregivers who consented to offer their time and contribute throughout the study time. We are grateful for data collectors, supervisors, food distributors, the health care providers, and all others who facilitated our work.

References

1. Black RE, Allen LH, Bhutta ZA, Caulfield LE, de Onis M, Ezzati M, et al. Maternal and child undernutrition: global and regional exposures and health consequences. Lancet 2008;371(9608):243–60.
2. Black RE, Victora CG, Walker SP, Bhutta ZA, Christian P, de Onis M, et al. Maternal and child undernutrition and overweight in low-income and middle-income countries. Lancet. 2013;382:427–51.
3. Nane D, Hatloy A, Tadesse E, Lindtjørn B. Research protocol local ingredients-based supplementary food as an alternative to corn-soya blends plus for treating moderate acute malnutrition among children aged 6 to 59 months: a randomized controlled non-inferiority trial in Wolaita. BMC Public Health. 2019;19(1689):1–9.
4. Tessema M, Belachewu T, Ersino G. Feeding patterns and stunting during early childhood in rural communities of Sidama, South Ethiopia. PAMJ. 2013;14(75):1–12.
5. Bliss J, Jensen N, Thiede B, Shoham J, Dolan C, Sibson V, Fenn B. Factors associated with the risk of acute malnutrition among children aged 6 to 36 months in households targeted by an emergency cash transfer program. Food Nutr Bull. 2016;37(3):387–400.
6. Manary MJ, Sandige HL. Management of acute moderate and severe childhood malnutrition. BMJ. 2008;337:1227–1230.
7. Medoua GN, Ntsama PM, Ndzana ACA, Essa'a VJ, Dimodi HT. Recovery rate of children with moderate acute malnutrition treated with ready-to-use supplementary food or improved corn-soya blend: a randomized controlled trial. Public Health Nutr. 2016;19(2):363–70.

8. Annan R, Webb P, Brown R. Management of moderate acute malnutrition (MAM): current knowledge and practice. CMAM Forum Technical Brief. 2014 Sep [cited 2020 Aug 15]. Available from https://www.ennonline.net/managementofmamcurrentknowledgeandpractice

9. World Health Organization (WHO). (2012). Technical note: supplementary foods for the management of moderate acute malnutrition in infants and children 6–59 months of age. Geneva: WHO.

10. Bhandari N, Mohan SB, Bose A, Iyengar SD, Taneja S, Mazumder S, et al. Efficacy of three feeding regimens for home-based management of children with uncomplicated severe acute malnutrition: a randomised trial in India. BMJ Global Health. 2016;1:e000144.

11. Amegovu AK, Ochola S, Ogwok P,Yiga, P, Musalima J, Juliana M. Efficacy of sorghum-peanut blend and corn-soy blend plus in the treatment of moderate acute malnutrition in children aged 6–59 months in Karamoja, Uganda: a cluster-randomized trial. Nutr Diet Suppl. 2014;6:75–84.

12. Central Statistical Agency. Ethiopian Demographic and Health Survey 2011. Addis Ababa, Ethiopia, and Calverton, Maryland, USA. 2012.

13. Isanaka S, Barnhart DA, McDonald CM, Ackatia-Armah RS, Kupka R, Doumbia S, et al. Cost-effectiveness of community-based screening and treatment of moderate acute malnutrition in Mali. BMJ Glob Health. 2019;4:1–10.

14. Prinzo ZW, Briend A. Dietary management of moderate malnutrition: time for a change. Food Nutr Bull. 2009;30(3):S265–6.

15. Golden MH. Proposed recommended nutrient densities for moderately malnourished children. Food Nutr Bull. 2009;30(3):S267–342.

16. Stobaugh H. Maximizing recovery and growth when treating moderate acute malnutrition with whey-containing supplements. Food Nutr Bull. 2018;39(2):S30–34.

17. Kennedy E, Branca F, Webb P, Bhutta Z, Brown R. Setting the scene: an overview of issues related to policies and programs for moderate and severe acute malnutrition. Food Nutr Bull. 2015;36(1):S9–14

18. James PT, Van den Briel N, Rozet A, Israe AD, Fenn B, Navarro-Colorado C. Low-dose RUTF protocol and improved service delivery lead to good programme outcomes in the treatment of uncomplicated SAM: a programme report from Myanmar. Matern Child Nutr. 2015;11:859–69.

19. Ashworth A, Ferguson E. Dietary counseling in the management of moderate malnourishment in children. Food Nutr Bull. 2009;30:S405–33.

20. Amegevu AK, Ogwok P, Ochola S, Yiga P, Musalima JH, Mutenyo E. Formulation of sorghum-peanut blend using linear programming for treatment of moderate acute malnutrition in Uganda. J Food Chem Nutr. 2013;1(2):67–77.

21. Karakochuk C, van den Briel T, Stephens D, Zlotkin S. Treatment of moderate acute malnutrition with ready-to-use supplementary food results in higher overall recovery rates compared with a corn-soya blend in children in southern Ethiopia: an operations research trial. Am J Clin Nutr. 2012; 96:911–6.

22. Ethiopia: Food insecurity revised emergency appeal no. MDRET005. 2008 [cited 2020 Aug 15]. Available from: https://reliefweb.int/report/ethiopia/ethiopia-food-insecurity-revised-emergency-appeal-no-mdret005

23. World Health Organization and United Nations Children's Fund. WHO child growth standards and the identification of severe acute malnutrition in infants and children. Geneva: WHO; 2009.

24. Ackatia-Armah RS, McDonald CM, Doumbia S, Erhardt JG, Hamer DH, Brown KH. Malian children with moderate acute malnutrition who are treated with lipid-based dietary supplements have greater weight gains and recovery rates than those treated with locally produced cereal-legume products: a community-based, cluster-randomized trial. Am J Clin Nutr. 2015;101:632–45.

25. LaGrone LN, Trehan I, Meuli GJ, Wang RJ, Thakwalakwa C, Maleta K, et al. A novel fortified blended flour, corn-soy blend “plus-plus,” is not inferior to lipid-based ready-to-use supplementary foods for the treatment of moderate acute malnutrition in Malawian children. Am J Clin Nutr. 2012;95:212–9.

26. The UN Refugee Agency and World Food Programme. Guidelines for selective feeding: the management of malnutrition in emergencies. Geneva, UNHCR; 2011.

27. Nikièma L, Huybregts L, Kolsteren, P, Lanou, H, Tiendrebeogo S, Bouckaert K, et al. Treating moderate acute malnutrition in first-line health services: an effectiveness cluster-randomized trial in Burkina Faso. Am J Clin Nutr. 2014;100,241–9.

28. Kaul S., Diamond GA. Good enough: a primer on the analysis and interpretation of noninferiority trials. Ann Intern Med. 2006;145:62–69.

29. Michaelsen KF, Hoppe C, Roos N, Kaestel P, Stougaard M, Lauritzen L, et al. Choice of foods and ingredients for moderately malnourished children 6 months to 5 years of age. Food Nutr Bull. 2009;30(3):S343–405.

30. Matilsky DK, Maleta K, Castleman T, Manary MJ. Supplementary feeding with fortified spreads results in higher recovery rates than with a corn/soy blend in moderately wasted children. J Nutr. 2009;139:773–8.

31. Nackers F, Broillet F, Oumarou D, Djibo A, Gaboulaud V, Guerin PJ, et al. Effectiveness of ready-to-use therapeutic food compared to a corn/soy-blend-based pre-mix for the treatment of childhood moderate acute malnutrition in Niger. J Trop Pediatr. 2010;56(6):407–13.

32. Lagrone L, Cole S, Schondelmeyer A, Maleta K, Manary MJ. Locally produced ready-to-use supplementary food is an effective treatment of moderate acute malnutrition in an operational setting. Ann Trop Paediatr. 2010; 30:103–8.

33. Patel MP, Sandige HL, Ndekha MJ, Briend A, Ashorn P, Manary MJ. Supplemental feeding with ready-to-use therapeutic food in Malawian children at risk of malnutrition. J Health Popul Nutr. 2005; 23:351–357.

34. Uauy R, Kurpad A, Tano-Debrah, Otoo GE, Aaron GA, Toride Y, et al. Role of protein and amino acids in infant and young child nutrition: protein and amino acid needs and relationship with child growth: J Nutr Sci Vitaminol. 2015;61:192–4.

35. Rivera JA, Hotz .C, Gonzalez-Cossio T, Neufeld L, Garcia-Guerra A. The effect of micronutrient deficiencies on child growth: a review of results from community-based supplementation trials. J Nutr Food Sci. 2003;133:S4010–20.

36. Stobaugh HC, Ryan.KN, Kennedy JA, Grise JB, Crocker AH, Thakwalakwa C, et al. Including whey protein and whey permeate in ready-to-use supplementary food improves recovery rates in children with moderate acute malnutrition: a randomized, double-blind clinical trial. Am J Clin Nutr. 2016;103:926–33.

Figure 1

Click here to access/download;Figure;Fig1TFR.tiff

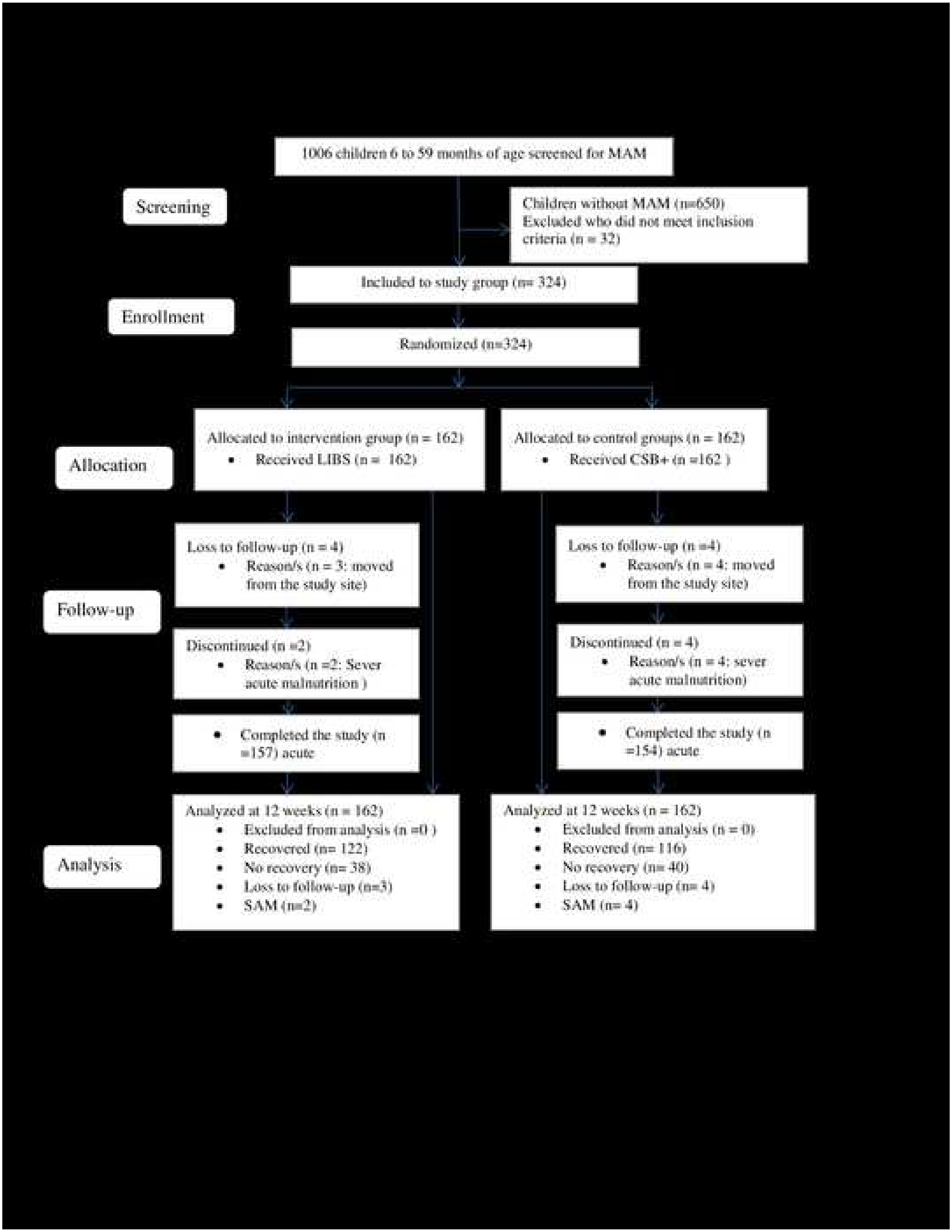

Figure 2

Click here to access/download;Figure;Fig2RR (2).tif

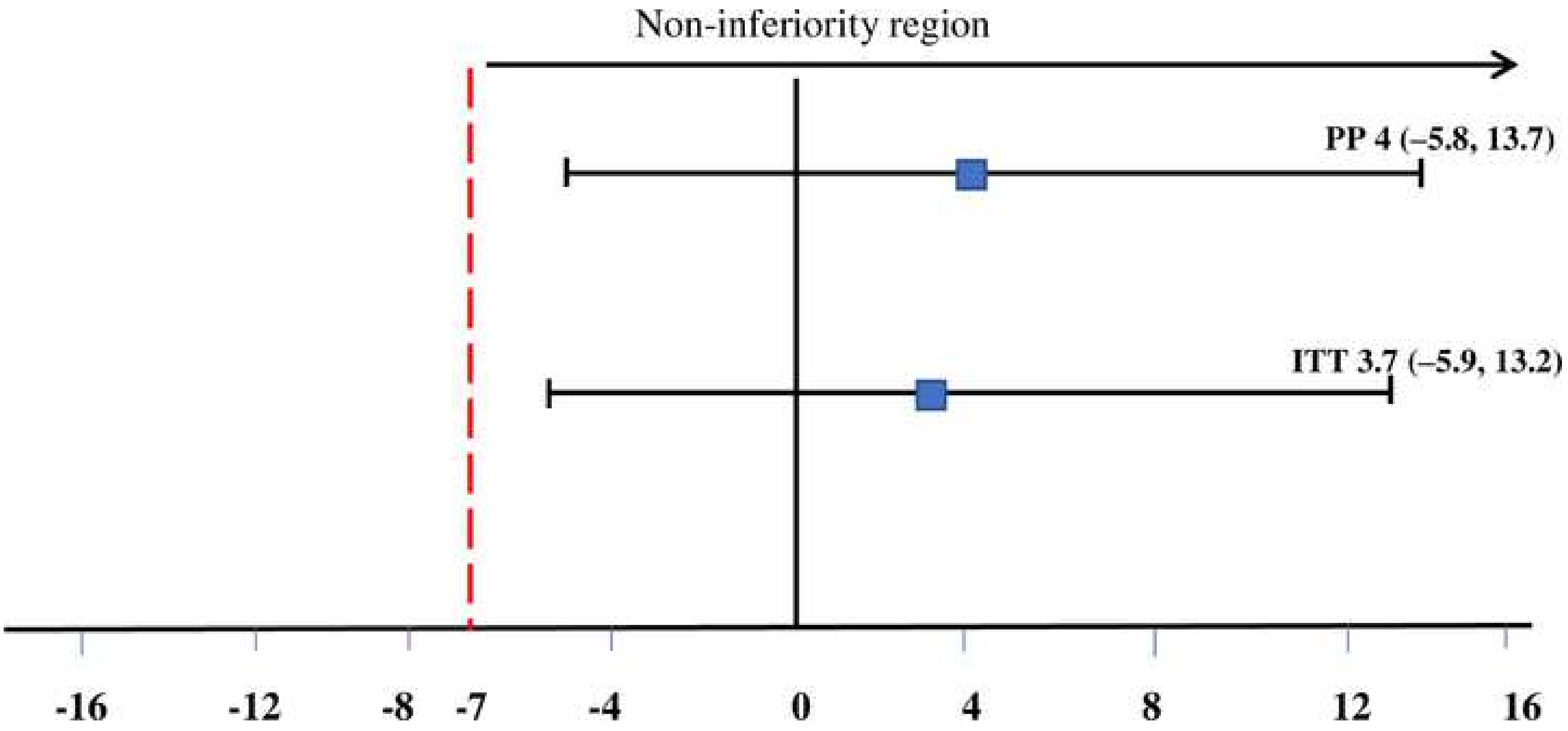

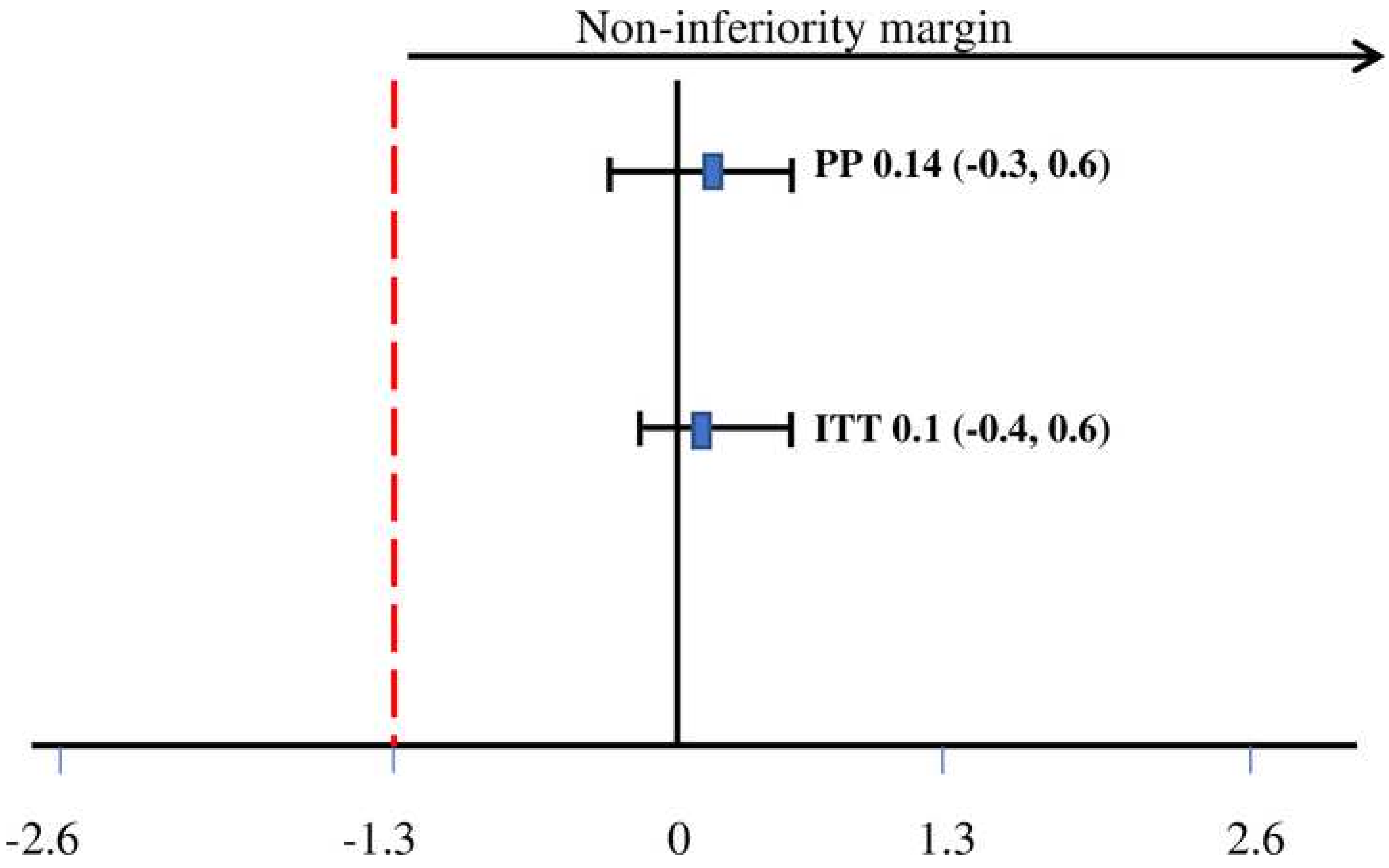
Non-inferiority margin
PP 0.14 (-0.3, 0.6)
ITT 0.1 (-0.4, 0.6)
-2.6
-1.3
0
1.3
2.6

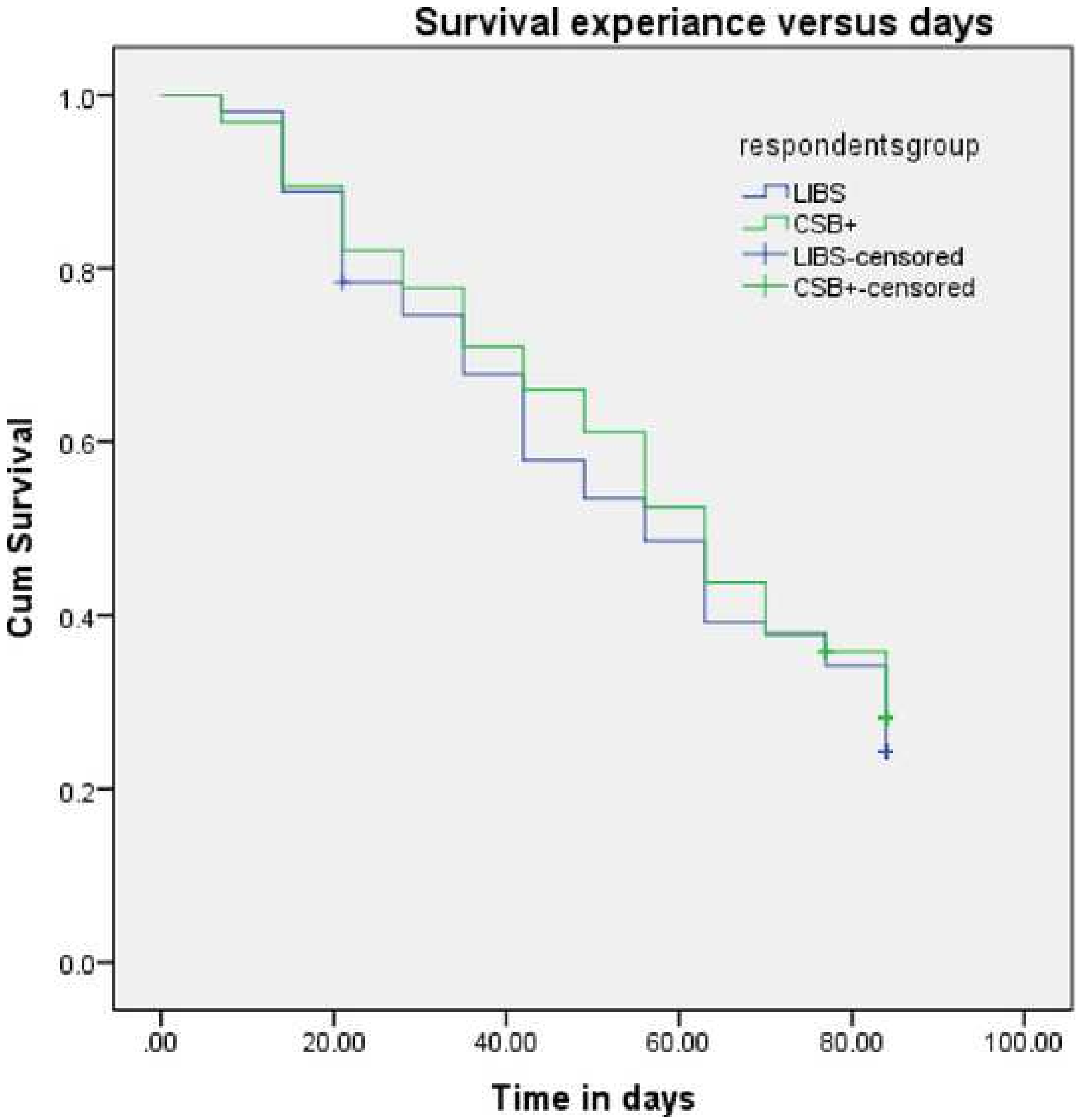
Survival experiance versus days
respondentsgroup
LIBS
CSB+
LIBS-censored
CSB+-censored
Cum Survival
1.0
0.8
0.6
0.4
0.2
0.0
.00
20.00
40.00
60.00
80.00
100.00
Time in days

Click here to access/download
Supporting Information
DN_RProtocol.docx

Click here to access/download
Supporting Information
CONSORT _DN.doc

Study protocol

Click here to access/download
Supporting Information
Study_protocol_DN.pdf
