# Supplementary material for: A local-ingredients-based supplement is an alternative to corn-soy blends plus for treating moderate acute malnutrition among children aged 6 to 59 months: A randomized controlled non-inferiority trial in Wolaita, Southern Ethiopia
Source: PLoS One. 2021 Oct 28;16(10):e0258715. doi: 10.1371/journal.pone.0258715 (PMC8553037; doi:10.1371/journal.pone.0258715)
Supplement: Attachment — Submitted filename: Response to reviewers_DN.docx [file pone.0258715.s005.docx]

**PLOS ONE**

**19.05.21**

**Dear Editor**

Thank you for allowing us to submit a revised manuscript titled ‘A local-ingredients-based supplement is an alternative to corn-soy blends plus for treating moderate acute malnutrition among children aged 6 to 59 months: A randomized controlled non-inferiority trial in Wolaita, Southern Ethiopia’ to *PLOS ONE* journal.

We are grateful to the editor and reviewers for their comments which allowed us to revise the manuscript. We have tried to improve the manuscript according to the comments raised by the reviewers.

For the reference of pages and line numbers, we used a track changed version of the manuscript. In this letter, the reviewers’ comments are written in bold format and our point-by-point responses are written in normal font. The change indicated in the manuscript is included in this letter and written in italics.

Yours sincerely,

Debritu Nane

Corresponding Author

**Review Comments to the Author**

**Reviewer #1**

**For Table 2, statistical testing to be avoided for baseline comparison between the two groups. Baseline statistical testing is only applicable to cluster randomization trials. Please refer to the CONSORT statement.**

Response: Statistical testing for baseline comparison between the LIBS and CSB+ groups was removed from the text and Table 2. The revision is found on page 18, line 381, page19, lines 390 to 391 (Table 2).

**Reviewer #2**

The format of the author's response is brutal and extremely difficult to read. I find it very difficult to know what has been added to the revised version because the additions are not indicated. In my view, the revisions I requested are necessary to improve the organization, readability and presentation of the material. Merely adding a few words, or referring to another part of the manuscript is not OK. If the authors are not willing to use the recommendations constructively, then the paper should be rejected.

Response: Thank you for your constructive comments and recommendations that allow us to revise our manuscript and apologies for the inconveniences while reading the previous response letter. We have tried to revise our manuscript and respond in a readable way. Responses for the comments are specified/tracked in the manuscript.

**Page 2 line 22 (in reviewed version): Define wasted scientifically, and/or replace in the abstract with something that malnourished**

Response: We have now used the terminology acute malnutrition throughout the paper. However, acute malnutrition means the same as wasting. This revisions are now found on page 4, lines 54, 55, 61, 65, and 71 (in revised version with track changes).

**Page 2 line 33 (in reviewed version): Is the sugar added to LIBS sucrose! Toxic**

Response: We have added cane sugar to the LIBS for two reasons. The first one is to improve the taste of LIBS. The second one is to improve or meet the calorie requirement that should come from carbohydrates, and to make it comparable with the CSB+. CSB+ is the conventional food provided for children with MAM in the control group. Regarding toxicity, the level of carbohydrates found in the LIBS is still lower than in CSB+.

This revision now reads as “*The cane sugar was added to the LIBS in which the taste of LIBS made better and the amount of calorie that should come from carbohydrate was improved but still lower than the level of carbohydrate found in the CSB+ (conventional food provided for children with MAM in the control group.”* This clarification is found in the revised manuscript with track changes on pages 14 to 15, lines 295 to 298 and page 15, line 309. Table 1

**Page 2 line 33 (in reviewed version): Specify oil**

Response: We included the revised sentence in the abstract part. We used refined deodorized and cholesterol-free sunflower oil.

The revised manuscript now reads as *“In the ﬁrst arm, 125.2 g of LIBS with 8 ml of refined deodorized and cholesterol-free sunflower oil/day was provided. In the second arm, 150 g of CSB+ with 16 ml of refined deodorized and cholesterol-free sunflower oil/day was provided*”. This revision is found on page 2, lines 32 to 35 (in revised version with track changes).

In the method section, we described the constituents of each intervention and specified what sugar and oil are.

The change made in the manuscript is as follows:

*“Subjects in the intervention group received a daily ration of 125.2 g of LIBS with 8 ml of refined deodorized and cholesterol free sunflower oil for 12 weeks. The composition of LIBS was: 30 g of pumpkin seed, 25 g of peanut grain, 20 g of amaranth grain, 15 g of flaxseed, 10 g of emmer wheat, and 25.2 g of cane sugar with 8 ml of refined deodorized and cholesterol free sunflower oil. This supplement (one serving) yielded 699 kcal, 22.6 g protein, 56.9 g carbohydrate, and 40.89 g fat. The cane sugar was added to the LIBS in which the taste of LIBS made better and the amount of calorie that should come from carbohydrate was improved but still lower than the level of carbohydrate found in the CSB+ (conventional food provided for children with MAM in the control group). Likewise, children in the control group received 150 g of CSB+/day with 16 ml of refined deodorized and cholesterol-free sunflower oil; this yielded 751 kcal, 21.25 g protein, 95 g carbohydrate, and 31.76 g fat daily for 12 weeks”.* This revised paragraph is found on pages 14 and 15, lines 290 to 301 (in revised version with track changes).

**Page 4 line 55 (in reviewed version): Explain what aspect of impaired growth and development is “acute”. Malnutrition must be chronic and or frequently repeated for impaired growth and development.**
Response: During acute malnutrition, there could be childhood infections that lead to death, debility and impairment in growth and development. When a repeated episode of acute malnutrition occurs, often in combination with infections, it can lead to stunting (chronic malnutrition). Sometimes, especially if the child is less than two years of age, it can also affected other developmental features such as brain impairment.

This information is added to the revised text with track changes on page 4, lines 61 to 64. It now reads as *“When a repeated episode of acute malnutrition occurs, often in combination with infections, it can lead to stunting (chronic malnutrition). Sometimes, especially if the child is less than two years of age, it can also affect other developmental features such as brain impairment”.*

**Page 4 line 63 (in reviewed version): Acute means what in terms of nutrient delivery?**

Response: Acute malnutrition is a recent thinness caused by inadequate intake of macronutrients (energy and protein), infections and other several factors. This information was added to the revised manuscript page 4, lines 55 to 58 (in revised version with track changes).

The information is as follows:

“*AM often occurs when there is severe weight loss possibly caused by inadequate energy and protein intake, and infections. Such events frequently occur during periods of prolonged food insecurity, poverty, poor feeding practices, and inadequate household food accessibility”.*

**Page 5 lines 86, 88 and 89 (in reviewed version): Specify oil and sugar found in CSB+ and CSB++**

Response: The specification of oil and sugar has now been added to the conventional food supplements (CSB+ and CSB++) for children with MAM were not mentioned in the literatures. Different literatures described the constituents of CSB, CSB+, and CSB++ but they did not specify which kind of sugars and oils that were used. The paragraph with the references is found in the manuscript, introduction part, page 5 and 6, lines 94 to 100 (in revised version with track changes). In our study, children with MAM got refined deodorized and cholesterol free sunflower oil during CSB+ ration but they may not be provided with sugar.

**Page 5 line 91 (in reviewed version): What cereal and grains that CSB+ made up of**

Response: CSB+ is a combination of the cereal (maize or corn) and a legume, most often soybean. The maize or corn found in CSB+ could be named as cereals and grains interchangeably.

The revised text now reads as “*The combinations of the cereal (corn or maize) and the legume (soybean) was refined, blended, and precooked (by roasting) for children with MAM below five years of age”*. This revision is found on page 6, lines 103 to 105 (in revised version with track changes).

**Page 7 line 120 (in reviewed version): Introduction needs better focus; reduce length by at least 30%; restrict to the introduction, not the discussion**

Response: To reduce the length of the introduction, we have removed some information from the text and moved into conclusion part. The revisions were indicated in the track changed manuscript of page 4, lines 58 to 61; pages 4,lines 71 to 73; page 5, lines 75 to 83 and lines 91 to 96; pages 6 and 7, lines 116 to 122, and page 7, lines 129 to 131 (in revised version with track changes).

Some information has also been moved to the conclusion part.

The revised and moved text is as follows:

*“The evidence drawn from this study will be shared with the public and with policymakers as LIBS has the potential to increase recovery from MAM and decrease the burden of malnutrition”.* This revision is found on page 27, lines 546 to 548 (in revised version with track changes).

**Page 7 line 121 (in reviewed version): Requirement of ethical statement at the beginning of methods part**

Response: The ethics approval and consent to participate statement is moved to the first part of the method section.

The paragraph in the revised manuscript is as follows:

## *“This study was approved by the Hawassa University College of Medicine and Health Sciences Institutional Review Board (IRB/024/10) and Regional Committees for Medical and Health Research Ethics in Norway (2018/69/REK vest).The approval obtained from the institutional review boards covered all sites included in the study. The purpose of the study and methods of data collection, confidentiality, and voluntary participation were explained to the mothers of children who were invited to sign an informed consent form.* *Verbal (getting thumb mark after reading the information) and written informed consent were obtained from all caregivers of children who met enrollment criteria before the recruitment of their children into the study. All interviews and intervention procedures were conducted in privacy. This trial was registered at Pan-African Clinical Trial Registration as PACTR201809662822990. The authors confirmed that all ongoing and related trials for these food supplements were registered”.* This revision is found on page 8, lines 140 to 149 (in revised version with track changes).

**Page 12 line 249 (in reviewed version): Provide details- specifics about the sugar and oil**

Response: We accepted the comment and specified the oil and sugar given to the subjects in the abstract and method parts. We used refined deodorized and cholesterol-free sunflower oil for both groups. Regarding sugar, we used refined cane sugar.

The revisions are as follows:

*“In the ﬁrst arm, 125.2 g of LIBS with 8 ml of refined deodorized and cholesterol-free sunflower oil/day was provided. In the second arm, 150 g of CSB+ with 16 ml of refined deodorized and cholesterol-free sunflower oil/day was provided”*. This revision is found in the abstract part page 2, lines 32 to 36 (in revised version with track changes).

*“This was a randomized, controlled, non-inferiority trial that assessed the efficacy of 125.2 g of LIBS with 8 ml of refined deodorized and cholesterol-free sunflower oil/day (the intervention), compared with conventional treatment, which is CSB+ in the amount of 150 g of CSB+/day with 16 ml of refined deodorized and cholesterol-free sunflower oil (the control), in treating MAM for 12 weeks”.* This revision is found in the method part under the study design on pages 9 and 10, lines 181 to 185 (in revised version with track changes).

In the method section under the description of the intervention, we have revised a paragraph.

Now it reads as “*Subjects in the intervention group received a daily ration of 125.2 g of LIBS with 8 ml of refined deodorized and cholesterol free sunflower oil for 12 weeks. The composition of LIBS was: 30 g of pumpkin seed, 25 g of peanut grain, 20 g of amaranth grain, 15 g of flaxseed, 10 g of emmer wheat, and 25.2 g of cane sugar with 8 ml of refined deodorized and cholesterol free sunflower oil. This supplement (one serving) yielded 699 kcal, 22.6 g protein, 56.9 g carbohydrate, and 40.89 g fat. The cane sugar was added to the LIBS in which the taste of LIBS made better and the amount of calorie that should come from carbohydrate was improved but still lower than the level of carbohydrate found in the CSB+ (conventional food provided for children with MAM in the control group). Likewise, children in the control group received 150 g of CSB+/day with 16 ml of refined deodorized and cholesterol-free sunflower oil; this yielded 751 kcal, 21.25 g protein, 95 g carbohydrate, and 31.76 g fat daily for 12 weeks.”* This revision is found on pages 14 to 15, lines 290 to 301 (in revised version with track changes).

**Page 12 line 251 (in reviewed version): Specify what flour is**

Response: Previously the sentence was written as follows: Children in the control group received CSB+ in the amount of 150 g flour/day with 16 ml of oil. We removed the term flour since it is unnecessary word.

Now it is revised as “*children in the control group received 150 g of CSB+/day with 16 ml of refined deodorized and cholesterol-free sunflower oil; this yielded 751 kcal, 21.25 g protein, 95 g carbohydrate, and 31.76 g fat daily for 12 weeks.* This revision is found on page 15, lines 298 to 301 (in revised version with track changes).

**Page 12 lines 250 and 252 (in reviewed version): The amount of carbs and ash in LIBS and CSB+**

Response: the amount of carbohydrate and ash found in both supplements is now mentioned in the revised manuscript. The amount of carbohydrate found in the LIBS is 56.9 g whereas the amount of carbohydrate found in the CSB+ is 95 g. The ash content in the CSB+ is higher (4.3g) than LIBS (2.1g).

These revisions are found on page 15, line 311 (in revised version with track changes). (Table 1)

*Here is the table.*

**Table 1. Nutrient composition of the supplementary foods.**

| Nutrient | 125 gm of LIBS with 8 ml oil | 150 gm of CSB+ with 16ml of oil |
| --- | --- | --- |
| Energy (kcl) | 698.5 | 750.84 |
| Protein (g) | 22.6 | 21.25 |
| Carbohydrate (g) | 56.9 | 95.0 |
| Fat (g) | 40.89 | 3176 |
| Ash (g) | 2.1 | 4.3 |
| Iron (mg) | 8.1 | 6.00 |
| Zn (mg) | 5.6 | 7.5 |
| Calcium (mg) | 100.00 | 195.00 |
| Phosphorous (mg) | 470.55 | 300.00 |
| Potassium (mg) | 666.14 | 600.00 |
| Magnesium (mg) | 394.7 | 107.75 |
| Sodium (mg) | 84.6 | 41.25 |
| Folic acid (µg) | 49.4 | 90.00 |

## **Major recommendations:**

**1. do a thorough job of defining MAM - it is incomplete**

Response: Previously, the definition of MAM was written in the text as “Moderate acute malnutrition (MAM) as weight-for-height z-score (WHZ) between -3 and -2 standard deviations and/or mid-upper arm circumference (MUAC) of between ≥11.5 cm and <12.5 cm”. To make the definition of MAM complete and differentiate it from severe form, we have added the information about edema.

The revised definition is as follows:

*“Moderate acute malnutrition (MAM) is defined as weight-for-height z-score (WHZ) between -3 and -2 z-scores of the WHO Child Growth Standards median and/or mid-upper arm circumference (MUAC) of between greater or equal to 11.5 cm and less than 12.5 cm, without bipedal edema”.* The reference is added to the manuscript. (This information is found on page 4, lines 65 to 68 in revised version with track changes).)

**2. do a thorough job of specifying what is meant by sugar, oil, vegetable oil - these are general terms.**

Response: The oil that we used for both groups was refined deodorized and cholesterol-free sunflower oil. The sugar that we used was cane sugar. We used cane sugar to improve the taste of LIBS and to make the amount of calories that should come from carbohydrates comparable with the CSB+ (conventional food provided for children with MAM in the control group).

The revisions that are found in the manuscript are as follows:

*“In the ﬁrst arm, 125.2 g of LIBS with 8 ml of refined deodorized and cholesterol-free sunflower oil/day was provided. In the second arm, 150 g of CSB+ with 16 ml of refined deodorized and cholesterol-free sunflower oil/day was provided.”* (This information is found on page 2, lines 32 to 35 in revised version with track changes)

*“This was a randomized, controlled, non-inferiority trial that assessed the efficacy of 125.2 g of LIBS with 8 ml of refined deodorized and cholesterol-free sunflower oil/day (the intervention), compared with conventional treatment, which is CSB+ in the amount of 150 g of CSB+/day with 16 ml of refined deodorized and cholesterol-free sunflower oil (the control), in treating MAM for 12 weeks”.* (This information is found on page 9 to 10, lines 181 to 185 in revised version with track changes)

“*Subjects in the intervention group received a daily ration of 125.2 g of LIBS with 8 ml of refined deodorized and cholesterol free sunflower oil for 12 weeks. The composition of LIBS was: 30 g of pumpkin seed, 25 g of peanut grain, 20 g of amaranth grain, 15 g of flaxseed, 10 g of emmer wheat, and 25.2 g of cane sugar with 8 ml of refined deodorized and cholesterol free sunflower oil. This supplement (one serving) yielded 699 kcal, 22.6 g protein, 56.9 g carbohydrate, and 40.89 g fat. The cane sugar was added to the LIBS in which the taste of LIBS made better and the amount of calorie that should come from carbohydrate was improved but still lower than the level of carbohydrate found in the CSB+ (conventional food provided for children with MAM in the control group). Likewise, children in the control group received 150 g of CSB+/day with 16 ml of refined deodorized and cholesterol-free sunflower oil; this yielded 751 kcal, 21.25 g protein, 95 g carbohydrate, and 31.76 g fat daily for 12 weeks.”* (Pages 14 to 15, lines 290 to 301 in revised version with track changes)

**3. focus the Introduction - some of the info here is better combined and condensed into the Discussion**

Response:

To reduce the length of the introduction, we have removed some information from the text and moved into conclusion part. The revisions were indicated in the track changed manuscript of page 4, lines 58 to 61; pages 4,lines 71 to 73; page 5, lines 75 to 83 and lines 91 to 96; pages 6 and 7, lines 116 to 122, and page 7, lines 129 to 131 (in revised version with track changes).

Some information has also been moved to the conclusion part.

The revised and moved text is as follows:

*“The evidence drawn from this study will be shared with the public and with policymakers as LIBS has the potential to increase recovery from MAM and decrease the burden of malnutrition”.* This revision is found on page 27, lines 546 to 548 (in revised version with track changes).

Reviewer #3: (No Response)

Reviewer #4: Well done on the changes, it made a significant difference to the quality of the paper. Please have a look for a few minor editing problems.
